# Modulating effect of emotional arousal intensity on selective attention in schizophrenia

**DOI:** 10.3389/fpsyt.2025.1593548

**Published:** 2025-09-01

**Authors:** Miaomiao Yu, Bo Dong, Feilong Qian, Jingfang Liu, Xiaogang Wu

**Affiliations:** ^1^ School of Education, Suzhou University of Science and Technology, Suzhou, China; ^2^ Suzhou Social Welfare General Hospital, Suzhou, China

**Keywords:** schizophrenia, inhibition of return, emotional arousal intensity, emotion, attention

## Abstract

**Introduction:**

The interaction between attention and emotion is one of the key questions in schizophrenia, but the mechanisms of how emotional information affects selective attention in schizophrenia are still unclear.

**Methods:**

The present study employed a cue-back-to-fixation procedure to manipulate the valence and emotional arousal intensity of stimuli presented at either cued locations (Experiment 1) or target locations (Experiment 2). The present study examined the impact of emotional arousal intensity on the inhibition of return (IOR)—a phenomenon characterized by faster responses to previously unattended relative to attended locations—in individuals with schizophrenia, during two distinct attentional phases: attentional disengagement and attentional reorientation.

**Results:**

The results showed significant IOR effects for both schizophrenia (Experiment 1a and 2a) and control groups (Experiment 1b and 2b) regardless of the emotional stimuli with different arousal intensities presented at both the cued and target locations. However, as compared with negative low arousal stimuli or neutral low arousal stimuli, significantly larger IOR effect size for control groups was found when negative high arousal stimuli were presented in cued location and for schizophrenia groups was found when negative high arousal stimuli were presented in target location.

**Discussion:**

These results may underly the mechanism of attentional deficit for schizophrenia towards different arousal intensities of emotional stimuli. During the attentional disengagement phase, schizophrenia patients are more likely to filtered out those high-arousal stimuli that endanger life while control group participants experience enhanced perceptual processing towards them; during the attentional reorientation phase, schizophrenia patients display “hyperfocusing” on those life-threatening high-arousal stimuli while the control group manifest an “attentional blindness” phenomenon to avoid these threatening stimuli. Meanwhile, we also interpreted our findings in light of an alternative theory of salience dysregulation.

## 1 Introduction

Schizophrenia represents a complex psychiatric disorder characterized by three core symptom clusters: positive symptoms, negative symptoms, and cognitive impairment, all of which significantly impair patients’ functional outcomes (1). Cognitive impairment in this population typically manifests through neuropsychological deficits in attention and memory and social cognitive impairments affecting intention processing and emotional recognition (2). Notably, attention deficits constitute a hallmark feature of schizophrenia, as evidenced by multiple empirical investigations (3–5). Researchers frequently employ spatial cueing paradigms to investigate these attentional abnormalities (6–8).

In the spatial cueing paradigm, a cued stimulus is first randomly presented to either the left or right side of a central fixation point. Following a variable time interval (stimulus onset asynchrony, SOA), a target stimulus appears with equal probability at either location (9). Experimental trials are classified as valid cue conditions when targets appear at the cued location, and invalid cue conditions when targets emerge at the uncued location. Empirical evidence reveals differential temporal patterns in attentional modulation: When SOA durations are less than about 300 ms, response times (RTs) under valid cue conditions demonstrate significant facilitation compared to invalid trials. However, this facilitatory effect undergoes a qualitative reversal at SOA intervals exceeding about 300 ms, with valid cue trials exhibiting slower RTs than invalid ones, a phenomenon termed inhibition of return (IOR) (10). Classical attentional re-orient theories propose a biphasic mechanism underlying these effects (11). Initial orientation involves rapid attentional engagement at the cued location, followed by subsequent disengagement processes. When targets reappear at previously attended locations, this sequence creates reorientation difficulties that paradoxically impair response efficiency. The temporal transition from facilitation to inhibition reflects adaptive mechanisms that prioritize novel spatial information during visual exploration.

Inconsistent results have been reported in studies utilizing the IOR effect to investigate attention deficits in schizophrenia (6, 12). Some studies have demonstrated normal IOR effects in individuals with schizophrenia (13–15), while others have found blunted or delayed IOR in this population (16–19). A meta-analysis suggested that inconsistencies in IOR findings among individuals with schizophrenia may be attributed to methodological differences (20). For instance, delayed IOR was observed in individuals with schizophrenia when using a single-cue procedure (without a central cue), whereas the time course of IOR was more consistent with healthy controls when a cue-back-to-fixation procedure (with a second cue, i.e., highlighting the central fixation point) was employed. In the study, the experimental design involved the presentation of three boxes and a central fixation point (19). The cue was displayed for 150 ms after the fixation point disappeared for 200 ms, followed by the presentation of a target after a variable SOA. The results revealed that individuals with schizophrenia exhibited slower overall responses compared to healthy participants, as well as delayed IOR. Similarly, researchers conducted two experiments to examine the IOR effect in schizophrenia (12). In Experiment 1, one of three boxes on the screen was randomly highlighted as the cue, and the target (an asterisk) appeared with equal probability in the left or right box after varying SOA. Experiment 2 replicated this design but included an additional central cue after the initially peripheral cue. The findings indicated that IOR was impaired in individuals with schizophrenia in the absence of a central cue but recovered when a central cue was introduced. Based on these studies, it can be inferred that the central cue facilitates the disengagement of attention from the cued location, calls attention back to the central fixation point, and reorients it toward the target stimulus. This suggests that the presence of a central cue may play a critical role in observing IOR effects in individuals with schizophrenia. However, it is crucial to note that attentional disengagement is not a necessary prerequisite for observing IOR (21, 22). IOR can still be detected even when attention remains sustained at the cued location. Furthermore, some researchers propose that central cues may interfere with the integrative processing of cues and target stimuli, thereby modulating IOR (23).

In addition to attention deficits, research on schizophrenia has also explored the interaction between emotion and attention. Emotional stimuli are more likely to attract attention compared to neutral stimuli (24, 25). Studies investigating IOR in schizophrenia have reported inconsistent findings when emotional stimuli are presented at either the cue or target location (6, 7, 18, 26). Specifically, researchers employed a single-cue procedure without a central cue and used threatening faces and scrambled faces (scrambled versions of threatening faces) as emotional stimuli to examine IOR in schizophrenia under variable stimulus onset asynchrony (SOA) conditions (18). In Experiment 1, an emotional face was used as the cue stimulus, and a small square served as the target. The results revealed no IOR effect in individuals with schizophrenia. In Experiment 2, a small square was used as the cue stimulus, and an emotional face was used as the target stimulus. Here, in individuals with schizophrenia, IOR was observed only when the target was a neutral scrambled face. Similar to research on schizophrenia, studies involving the general population have also focused on the interaction between emotion and attention. When emotional stimuli are presented at the cue location, some studies have found that individuals struggle to disengage attention from negative emotional stimuli, leading to a weakened IOR effect (11, 27–30). Conversely, other studies have reported that negative emotional cues can enhance the IOR effect (31–33). However, some studies have found no significant differences in IOR between negative and neutral cues (34, 35). When emotional stimuli are presented at the target location, some studies have reported a reduced IOR effect size in the presence of negative emotional stimuli (36), while others found that the emotional characteristics of the target stimulus did not influence IOR (34). Researchers suggested that these inconsistencies may be related to the intensity of emotional arousal (32). They used negative high arousal, neutral, and scrambled emotional stimuli as cues and asked participants to perform a simple detection task for the target. They found that negative high arousal cues produced a greater IOR effect compared to neutral or scrambled cues. The researchers proposed that highly arousing negative stimuli enhance the perceptual processing of spatial cues, and this enhanced processing may be linked to the evolutionary advantage of effectively disengaging attention from threatening stimuli (32). In another study of psychopathology, researchers also found the modulating effect of emotional arousal intensity on IOR. Based on these findings, it is suggested that research has primarily focused on the influence of emotional stimuli on IOR when presented at either the cue or target location, with the arousal level of emotional stimuli emerging as a significant influencing factor. However, current research on schizophrenia has yet to systematically explore the impact of emotional arousal intensity.

Previous studies have demonstrated that the intensity of emotional arousal can significantly influence cognitive processing, with high arousal emotional stimuli attracting greater attentional resources (24, 25). Researchers utilized a spatial cueing paradigm to investigate the effects of emotional valence and arousal levels on the allocation of spatial attention (37). In their experiment, emotional pictures varying in valence and arousal levels were used as cues, and a small black square served as the target. The results indicated that shifting spatial attention away from high arousal stimulus pictures was slower compared to low arousal stimulus pictures, while the valence of the emotional cues had no significant effect. These findings support the notion that emotional arousal plays a critical role in attentional allocation. Further research has revealed that the relationship between emotional arousal and memory performance follows an inverted U-shaped curve, with moderate levels of emotional arousal facilitating better memory retention (38). Researchers examined the impact of emotional arousal on false memories and found that high arousal emotions, regardless of their positivity or negativity, produced more false memories compared to low arousal emotions (39). They also noted that higher arousal levels enhanced the processing of central information while reducing the accuracy of peripheral information. Additionally, studies have shown that emotional arousal levels modulate not only height perception (40) but also the intensity of emotional perception (41, 42). These findings collectively highlight the pervasive influence of emotional arousal on various cognitive and perceptual processes.

Based on these findings, current study employs a cue-back-to-fixation paradigm to examine the mechanisms through how emotional arousal intensity modulates IOR in schizophrenia patients as compared with control groups. Previous studies examining the impact of emotional stimuli on IOR have primarily focused on differences in IOR effects when emotional stimuli are presented at either the cue location or the target location. As highlighted, the arousal intensity of emotional stimuli may be a critical factor modulating the IOR effect (32). To explore this further, the present study presents negative emotional stimuli with varying arousal intensities at the cued location (Experiment 1) and the target location (Experiment 2). This design allows for an examination of how the arousal intensity of emotional stimuli influences IOR in schizophrenia during two distinct attentional phases: the attentional disengagement phase and the attentional reorient phase.

## 2 Experiment 1 emotional stimuli presented in the cued location

In Experiment 1, we investigated the impact of cues with varying emotional arousal intensities on IOR in schizophrenia patients and control group participants. Thus, Experiment 1 was divided into two sub-experiments: Experiment 1a involved schizophrenia groups while Experiment 1b comprised healthy control groups. The only difference between the two experiments was the participants, with all other aspects being the same.

### 2.1 Methods

#### 2.1.1 Participants

##### 2.1.1.1 Experiment 1a

Twenty-seven individuals diagnosed with schizophrenia were recruited from Suzhou Social Welfare General Hospital to participate in this study. All participants met the diagnostic criteria for schizophrenia as outlined in the Diagnostic and Statistical Manual of Mental Disorders, 5th Edition (DSM-5). At least two experienced psychiatrists evaluated each participant’s symptoms through structured clinical interviews. Exclusion criteria included: (1) a history of drug abuse or dependence within six months before the experiment; (2) a history of head injury resulting in persistent loss of consciousness or neurological sequelae; (3) cerebral metabolic abnormalities caused by neurological or other medical conditions. All participants reported no visual impairments and were able to complete the experiment without difficulty. Importantly, participants were unaware of the study’s purpose. Data from all 27 participants were included in the final analysis, with detailed demographic and clinical characteristics provided in **Table 1**. To evaluate the statistical power of this study, a sensitivity analysis was conducted using a two-sided paired samples t-test in G*Power 3.1.9.7 (43, 44). The analysis was performed with an alpha level of 0.05 and a power of 0.80 (45). The calculated effect size was *Cohen’s d* = 0.56, which is considered a medium effect size and indicates adequate statistical power based on established guidelines (46). This sensitivity analysis is based on *post-hoc* comparisons with Bonferroni correction for IOR effect under different arousal intensities.

**Table 1:** Basic information of participants.

Group	Characteristics	Male	Female
Schizophrenia	Age	55.33±8.09	52.17±12.63
	Duration of illness	31.07±9.22	26.50±10.47
Control	Age (Experiment 1)	23.08±2.07	20.05±1.51
	Age (Experiment 2)	23.57±2.21	20.09±2.31

The schizophrenia group consisted of the same 27 participants (15 males, 12 females) across both Experiment 1 and Experiment 2. The control group comprised 31 participants in Experiment 1 (12 males, 19 females) and 31 participants in Experiment 2 (14 males, 17 females).

##### 2.1.1.2 Experiment 1b

The control group consisted of 31 participants recruited from the campus community, all with normal or corrected-to-normal vision. Participants were naive to the experimental purpose and provided verbal or written informed consent. The calculated effect size was *Cohen’s d* = 0.52, indicating a medium effect size (46).

#### 2.1.2 Apparatus and materials

The experiment was programmed, and data were recorded using PsychoPy (https://www.psychopy.org/). All stimuli were displayed on a 22-inch ViewSonic P225f monitor with a resolution of 1024 × 768 pixels and a refresh rate of 100 Hz. The background of the experiment was set to grey. A central fixation point, represented by a “+” symbol, was displayed at the center of the screen (0, 0), with horizontal and vertical visual angles of 0.5°. The cued central fixation point, a bold “+” symbol, had horizontal and vertical visual angles of 1°. Two peripheral boxes, each with a horizontal visual angle of 5.3° and a vertical visual angle of 4°, were positioned to the left (-4°, 0°) and right (4°, 0°) of the fixation point, respectively (coordinates were defined with the central fixation point as the origin, measured in degrees of visual angle). The target stimulus was a white five-pointed star with horizontal and vertical visual angles of 3°. During the experiment, participants’ heads were stabilized using a chin rest to maintain a consistent viewing distance of approximately 60 cm from the center of the display screen.

The emotional stimuli used in the experiment were selected from the International Affective Picture System (IAPS) (47), developed by the Center for Research on Emotion and Attention at the National Institute of Mental Health (NIMH) in the United States. Based on the valence and arousal dimensions of the pictures, 10 negative high arousal pictures, 10 negative low arousal pictures, and 10 neutral low arousal pictures were selected for the experiment. The valence and arousal scores for each category of pictures are presented in **Table 2**. For emotional valence, a significant main effect was found, *F*(2,18) = 111.59, *p* < 0.001, *η^2^* = 0.93; pairwise comparisons found a significant difference between negative high arousal and neutral low arousal [*t*(9) = -10.24, *p* < 0.001], negative low arousal and neutral low arousal [*t*(9) = -20.34, *p* < 0.001], but no significant difference between negative high arousal and negative low arousal [*t*(9) = 0.22, *p* > 0.05]. For emotional arousal, a significant min effect was found, *F*(2,18)=131.97, *p*<0.001, *η^2^* = 0.94; pairwise comparisons found a significant difference between negative high arousal and negative low arousal [*t*(9) = 58.56, *p* < 0.001] and negative high arousal and neutral low arousal [*t*(9) = 12.43, *p* < 0.001], but no significant difference between negative low arousal and neutral low arousal [*t*(9) = 2.03, *p* > 0.05]. Negative pictures included images of mutilated corpses, weapons, violent scenes, and threatening animals, while neutral pictures depicted landscapes, objects, and similar content. Participants did not exhibit abnormal reactions or fear toward the negative stimulus pictures and were able to complete the experiment without difficulty.

**Table 2:** Valence and arousal scores of different types of emotional pictures in Experiment 1 and Experiment 2.

Dimensions of Emotion	Experiment 1 and Experiment 2		
	Negative high arousal	Negative low arousal	Neutral low arousal
Valence	2.84±0.59	2.80±0.12	5.13±0.36
Arousal	6.96±0.16	4.46±0.12	3.97±0.78

#### 2.1.3 Experimental design and experimental procedure

This experiment employed a 2 (cue validity: valid vs. invalid) × 3 (intensity of emotional arousal: negative high arousal vs. negative low arousal vs. neutral low arousal) within-subjects design. The dependent variable was response time.

**Figure 1:**
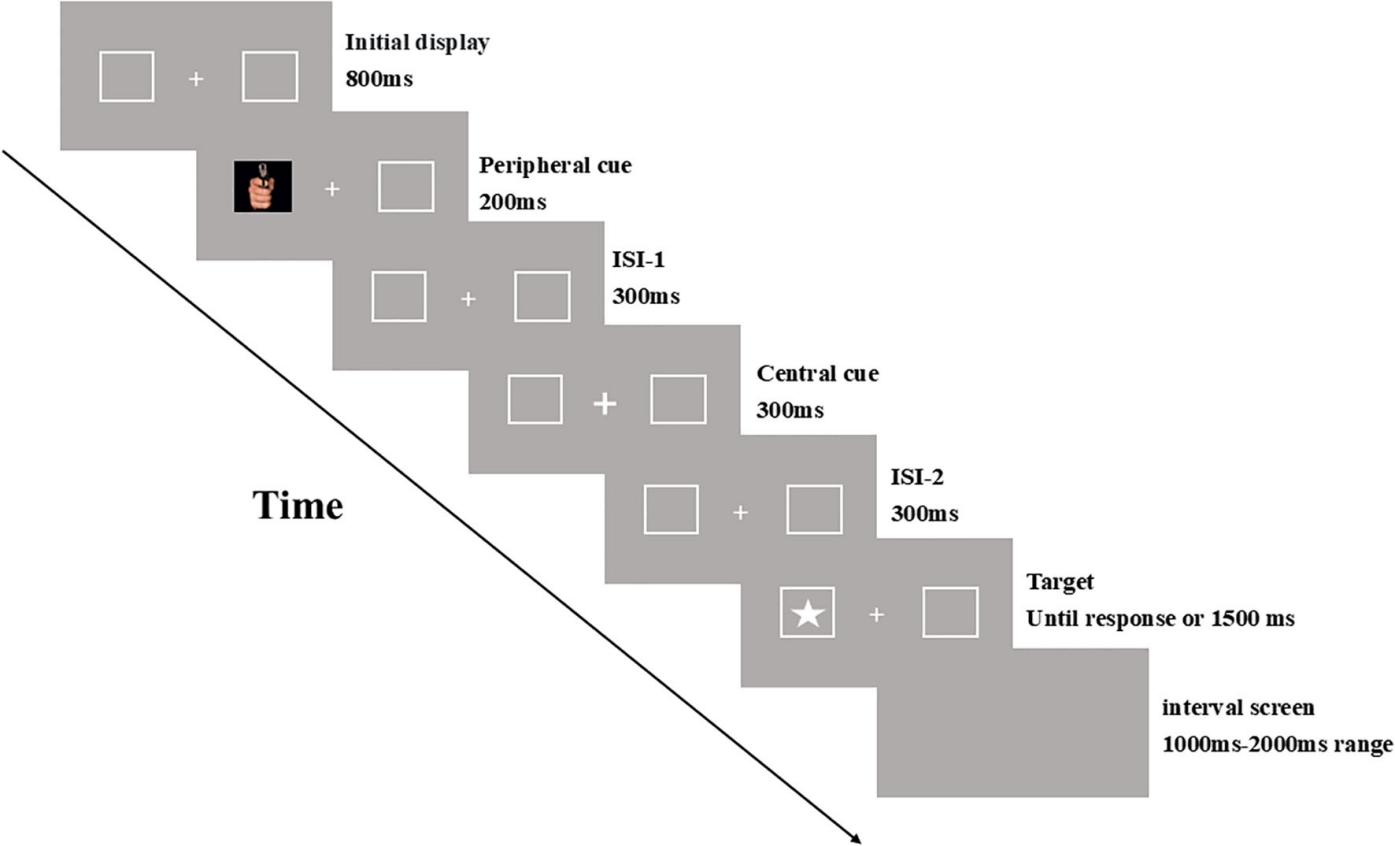
Experiment 1 procedure.

The experimental procedure is illustrated in **Figure 1**. Each trial began with the presentation of a fixation point (“**+**”) at the center of the screen, flanked by a rectangular box on each side. After 800 ms, emotional stimulus pictures with varying arousal intensities were randomly displayed in one of the rectangular boxes as peripheral cues for 200 ms. Following a 300 ms interval, the central fixation point (“+”) was replaced by a bolded central cue (“**+**”), which reverted to the original fixation point after another 300 ms. After an additional 300 ms, the target stimulus (a white five-pointed star) appeared randomly in either the left or right rectangular box. Participants were instructed to press the “G” key as quickly and accurately as possible upon detecting the target. After a response was made or 1500 ms had elapsed, an interval screen was displayed for a random duration ranging from 1000 ms to 2000 ms, after which the next trial began.

To familiarize participants with the experimental procedure and ensure they understood the tasks, each participant completed a practice session consisting of 20 trials before the formal experiment. Among these practice trials, two were catch trials, in which only the emotional stimulus pictures (cues) were presented without the target. The formal experiment comprised 264 trials, including 24 catch trials where the target did not appear, and participants were instructed not to press any key. In the remaining trials, all levels of the two independent variables — cue validity (valid vs. invalid) and emotional arousal intensity (negative high arousal, negative low arousal, and neutral low arousal) — were presented in a randomized, mixed order. Valid and invalid cues were equally probable, with negative high arousal, negative low arousal, and neutral low arousal stimuli presented with equal probability across these two conditions. The formal experiment was divided into two blocks, with participants allowed a brief one-minute rest between blocks.

### 2.2 Results

The mean error rates for Experiment 1a and Experiment 1b under different emotional conditions were presented in **Table 3**.

**Table 3:** Descriptive statistics of participants' error rates under different emotional conditions.

Emotional conditions		Control		Schizophrenia	
		Experiment 1b	Experiment 2b	Experiment 1a	Experiment 2a
Negative high arousal		0.0028	0.0012	0.05	0.04
Negative low arousal		0.0028	0.0012	0.05	0.04
Neutral low arousal		0.0024	0.0008	0.04	0.05

For each participant, trials with incorrect responses and those exceeding three standard deviations above or below the mean were excluded. Subsequently, individual mean reaction times under different experimental conditions were calculated. A two-factor repeated-measures analysis of variance (ANOVA) with 2 (cue validity: valid vs. invalid) × 3 (emotional arousal intensity: negative high arousal, negative low arousal, neutral low arousal) was conducted on the average reaction times using SPSS 25.0.

#### 2.2.1 Experiment 1a

**Figure 2:**
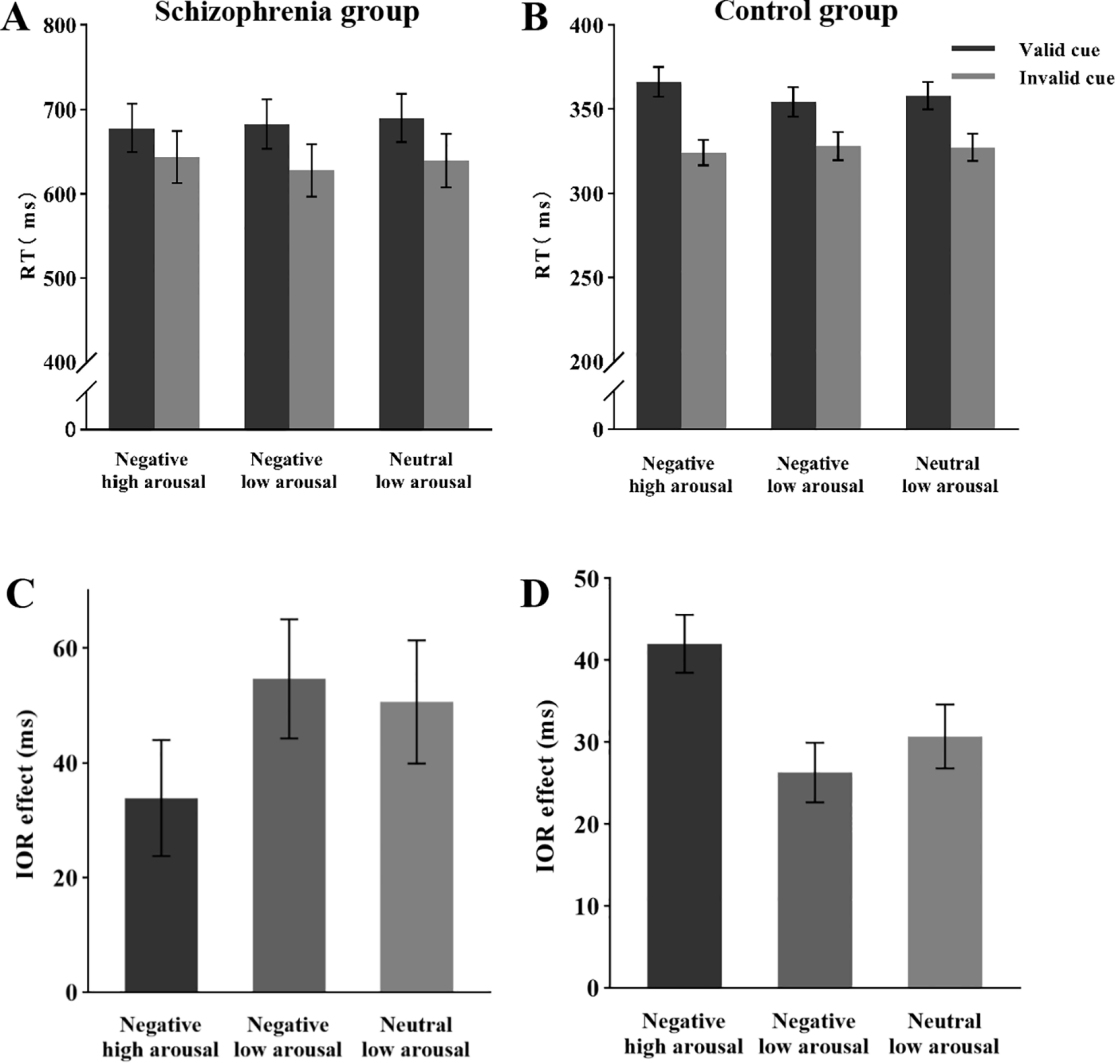
Mean RT and IOR under different conditions during the attentional disengagement phase. **(A)** Mean RT of schizophrenia group under different conditions. **(B)** Mean RT of the control group under different conditions. **(C)** IOR of schizophrenia group under different conditions. **(B)** IOR of the control group under different conditions.

Analysis of the mean reaction times (**Figure 2A**) revealed a significant main effect of cue validity, *F* (1, 26) = 34.24, *p* < 0.05, *η_p_^2^* = 0.57, with longer response times in the valid cue condition (*M* = 683 ms) compared to the invalid cue condition (*M* = 637 ms). The main effect of emotional arousal intensity was not significant, *F* (2, 52) = 1.53, *p* = 0.23, *η_p_^2^* = 0.05. Additionally, the interaction between cue validity and emotional arousal intensity was not significant, *F* (2, 52) = 1.79, *p* = 0.18, *η_p_^2^* = 0.06. Analysis of the IOR effect size (**Figure 2C**) revealed that the main effect of emotional arousal intensity was not significant, *F* (2, 52) = 1.79, *p* = 0.18, *η_p_^2^* = 0.06.

#### 2.2.2 Experiment 1b

Analysis of the mean reaction times (**Figure 2B**) revealed a significant main effect of cue validity, *F* (1, 30) = 106.49, *p* < 0.05, *η_p_^2^* = 0.78, with longer reaction times in valid cue conditions (359 ms) compared to invalid cue conditions (326 ms). The main effect of emotional arousal intensity was not significant, *F* (2, 60) = 2.43, *p* = 0.10, *η_p_^2^* = 0.08. A significant interaction emerged between cue validity and emotional arousal intensity, *F* (2, 60) = 12.57, *p* < 0.05, *η_p_^2^* = 0.30. *Post hoc* comparisons with Bonferroni correction demonstrated: Under negative high-arousal conditions, significantly longer reaction times in valid trials (366 ms) versus invalid trials (324 ms), *F* (1, 30) = 141.28, *p* < 0.001, *η_p_^2^* = 0.83, with the IOR effect size of 42 ms. In negative low-arousal conditions, valid trials (354 ms) showed significantly prolonged responses compared to invalid trials (328 ms), *F* (1, 30) = 51.55, *p* < 0.001, *η_p_^2^* = 0.63, with the IOR effect size of 26 ms. For neutral low-arousal conditions, valid trials (358 ms) exhibited significantly slower responses than invalid trials (327 ms), *F* (1, 30) = 61.74, *p* < 0.001, *η_p_^2^* = 0.67, with the IOR effect size of 31 ms.

Analysis of the IOR effect size (**Figure 2D**) revealed a significant main effect of emotional arousal intensity, *F* (2, 60) = 12.57, *p* < 0.05, *η_p_^2^* = 0.30. *Post hoc* comparisons with Bonferroni correction demonstrated: The IOR effect size for negative high-arousal stimuli (42 ms) was significantly greater than that for negative low-arousal stimuli (26 ms), *t* (60) = 4.86, *p* < 0.05, *d* = 0.76, *95% CI* [7.34, 24.14]. The IOR effect size for negative high-arousal stimuli (42 ms) significantly exceeded that for neutral low-arousal stimuli (31 ms), *t* (60) = 3.50, *p* < 0.05, *d* = 0.55, *95% CI* [3.18, 19.48]. No significant difference emerged between negative low-arousal (26 ms) and neutral low-arousal (31 ms) conditions, *t* (60) = -1.36, *p* = 0.53, *d* = -0.21, *95% CI* [-12.49, 3.67].

### 2.3 Discussion

The results of Experiment 1 revealed a significant main effect of cue validity in schizophrenia and control groups, with slower response times in the valid cue condition compared to the invalid cue condition. This finding suggests that a significant IOR effect was observed across all conditions. However, the interaction between cue validity and emotional arousal intensity was not significant in schizophrenia. This indicates that, under different cue-validity conditions, there were no differences in the IOR effect in individuals with schizophrenia, regardless of whether negative or neutral emotional cues were presented.

## 3 Experiment 2 emotional stimuli presented in the target location

In Experiment 2, we investigated the impact of targets with varying emotional arousal intensities on IOR in schizophrenia patients and control group participants. Thus, Experiment 2 was divided into two sub-experiments: Experiment 2a involved schizophrenia groups while Experiment 2b comprised healthy control groups. The only difference between the two experiments was the participants, with all other aspects being the same.

### 3.1 Methods

#### 3.1.1 Participants

##### 3.1.1.1 Experiment 2a

The 27 participants in the schizophrenia group for Experiment 2a were identical to those in Experiment 1a. To control for order effects, approximately half of the participants completed Experiment 1a followed by Experiment 2a, while the other half underwent the reverse sequence.

##### 3.1.1.2 Experiment 2b

The control group in Experiment 2b consisted of 31 participants, with 9 individuals differing from those in Experiment 1b (see **Table 1** for demographic details). The experimental order for participants who completed both experiments was counterbalanced.

#### 3.1.2 Apparatus and materials

The same as those in Experiment 1.

#### 3.1.3 Experimental design and experimental procedure

This experiment adopted a two-factor within-subjects design of 2 (cue validity: valid vs. invalid) × 3 (emotional arousal intensity: negative high arousal vs. negative low arousal vs. neutral low arousal), with the reaction time as the dependent variable.

The procedure of Experiment 2 was similar to that of Experiment 1. The difference was that in Experiment 2, the white five-pointed star was used as the peripheral cue, and the emotional stimulus pictures with different arousal intensities were used as the targets.

### 3.2 Results

The error rates for Experiment 2a and Experiment 2b under different emotional conditions are presented in **Table 3**. The analytical methods for mean reaction times and IOR effect size were identical to those employed in Experiment 1.

#### 3.2.1 Experiment 2a

**Figure 3:**
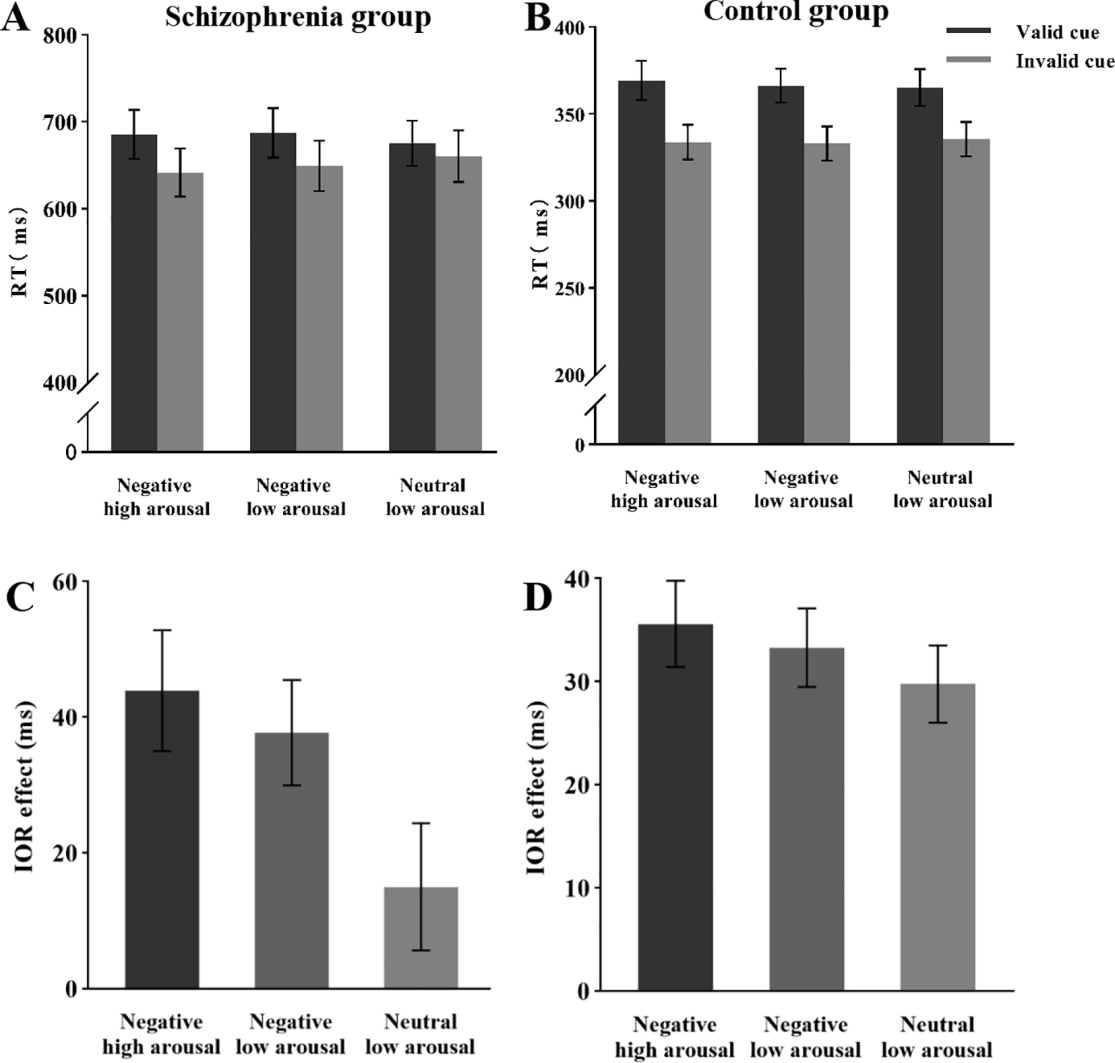
Mean RT and IOR under different conditions during the attentional reorientation phase. **(A)** Mean RT of schizophrenia group under different conditions. **(B)** Mean RT of the control group under different conditions. **(C)** IOR of schizophrenia group under different conditions. **(D)** IOR of the control group under different conditions.

Analysis of the mean reaction times (**Figure 3A**) revealed a significant main effect of cue validity, *F* (1, 26) = 23.41, *p* < 0.05, *η_p_^2^* = 0.47, with longer response times in the valid cue condition (*M* = 683 ms) compared to the invalid cue condition (*M* = 651 ms). The main effect of emotional arousal intensity was not significant, *F* (2, 52) = 0.74, *p* = 0.48, *η_p_^2^* = 0.03. However, a significant interaction was observed between cue validity and emotional arousal intensity, *F* (2, 52) = 4.92, *p* < 0.05, *η_p_^2^* = 0.16. *Post hoc* comparisons with Bonferroni correction demonstrated: Under the negative high arousal condition, participants’ reaction times in valid trials (*M* = 686 ms) were significantly slower than those in invalid trials (*M* = 642 ms), *F* (1, 26) = 24.41, *p* < 0.001, *η_p_^2^* = 0.48, with the IOR effect size of 44 ms. Under the negative low arousal condition, reaction times in valid trials (*M* = 687 ms) were significantly slower than those in invalid trials (*M* = 650 ms), *F* (1, 26) = 23.65, *p* < 0.001, *η_p_^2^* = 0.47, with an IOR effect size of 37 ms. In contrast, under the neutral low arousal condition, there was no significant difference between reaction times in valid trials (*M* = 675 ms) and invalid trials (*M* = 660 ms), *F* (1, 26) = 2.56, *p* = 0.12, *η_p_^2^* = 0.09, with an IOR effect size of 15 ms (not significant).

Analysis of the IOR effect size (**Figure 3C**) revealed a significant main effect of emotional arousal intensity, *F* (2, 52) = 4.92, *p* < 0.05, *η_p_^2^* = 0.16. *Post hoc* comparisons with Bonferroni correction indicated that the IOR effect size for negative high arousal emotional targets (*M* = 44 ms) was significantly larger than that for neutral low arousal emotional targets (*M* = 15 ms), *t* (52) = 2.98, *p* < 0.05, *d* = 0.64, 95% CI [1.58, 56.25]. However, there was no significant difference in the IOR effect size between negative high arousal (*M* = 44 ms) and negative low arousal (*M* = 38 ms) conditions, *t* (52) = 0.64, *p* = 1.00, *d* = 0.14, 95% CI [-16.92, 29.33]. Similarly, no significant difference was found between negative low arousal (*M* = 38 ms) and neutral low arousal (*M* = 15 ms) conditions, *t* (52) = 2.43, *p* = 0.67, *d* = 0.50, 95% CI [-1.11, 46.53].

#### 3.2.2 Experiment 2b

Analysis of the mean reaction times (**Figure 3B**) revealed a significant main effect of cue validity, *F* (1, 30) = 99.12, *p* < 0.05, *η_p_^2^* = 0.77, with longer reaction times in valid cue conditions (367 ms) compared to invalid cue conditions (334 ms). The main effect of emotional arousal intensity was not significant, *F* (2, 60) = 0.42, *p* = 0.66, *η_p_^2^* = 0.01. No significant interaction was observed between cue validity and emotional arousal intensity, *F* (2, 60) = 1.29, *p* = 0.28, *η_p_^2^* = 0.04. Analysis of the IOR effect size (**Figure 3D**) revealed that the main effect of emotional arousal intensity was not significant, *F* (2, 60) = 1.29, *p* < 0.05, *η_p_^2^* = 0.04.

### 3.3 Discussion

The results of Experiment 2 revealed a significant main effect of cue validity in schizophrenia and control groups, with participants responding faster in the invalid cue condition compared to the valid cue condition. This finding is consistent with the results of Experiment 1, indicating a significant IOR effect. We propose that the presence of a central cue in both experiments facilitated the occurrence of IOR. The control group showed no significant differences in the effect size of IOR across the three emotional conditions. However, a significant interaction was observed between cue validity and emotional arousal intensity, suggesting that the influence of cue validity on IOR in individuals with schizophrenia varies significantly depending on the intensity of emotional arousal. Specifically, the effect size of IOR was significantly larger for negative high arousal emotional targets (*M* = 44 ms) compared to neutral low arousal emotional targets (*M* = 15 ms). This enhancement of the IOR effect by negative high arousal emotional targets may be attributed to the heightened sensitivity of individuals with schizophrenia to negative emotions, as well as the characteristics of high arousal emotions that amplify their processing of target stimuli.

## 4 General discussion

Using the cue-back-to-fixation procedure, which included a central cue, the present study investigated the effects of emotional arousal intensity on IOR in individuals with schizophrenia and healthy participants. This was achieved by manipulating the arousal levels of emotional stimuli presented at the cue location (Experiment 1) and the target location (Experiment 2), respectively, to examine their influence during the attentional disengagement phase and the attentional reorientation phase. The results showed significant IOR effects in both Experiment 1a/2a (schizophrenia) and Experiment 1b/2b (control). In Experiment 1a, no significant differences in IOR effect size were observed across emotional stimulus conditions. Experiment 1b demonstrated significantly larger IOR effect size under negative high arousal conditions compared to both negative low arousal and neutral low arousal conditions. Experiment 2a exhibited significantly greater IOR effect size for negative high arousal stimuli compared to neutral low arousal stimuli or negative low arousal stimuli. Experiment 2b showed no significant differences in IOR effect size across emotional stimulus conditions.

In this study, a significant IOR effect was observed in individuals with schizophrenia, regardless of whether emotional stimuli were presented as cues or targets. However, researchers found only a weakened IOR effect in schizophrenia under neutral conditions and did not observe an IOR effect for emotional stimuli (18). We propose that this discrepancy in results is likely due to differences in experimental paradigms, specifically the presence or absence of central cues. They employed a single-cue procedure without central cues (18), whereas our experiments utilized the cue-back-to-fixation procedure, which included central cues. The presentation of a central cue after the initial cue effectively facilitated the disengagement of attention from the cued location in individuals with schizophrenia, thereby inhibiting stimuli presented at the cued location and ultimately helping to overcome deficits in IOR (16, 17, 20). Supporting this, researchers found that individuals with schizophrenia exhibited a significant IOR effect after the presentation of a second central cue designed to redirect attention (12). The study concluded that while there is no inherent deficit in attentional disengagement in schizophrenia, the effective return of attention to the central location is critical for observing IOR. Without this reorientation, the facilitation effect of the cued location may prevent the IOR from being observed. In contrast, studies using the single-cue procedure have reported that individuals with schizophrenia frequently revisited previously cued locations, with visual exploration confined to the cued area, resulting in either no IOR or a diminished IOR effect (19, 48). Based on these findings, the present study suggests that the inclusion of a central cue is a critical factor in enabling individuals with schizophrenia to exhibit the IOR effect.

In Experiment 1a, the interaction between cue validity and emotional arousal intensity was not significant in schizophrenia. Under conditions of varying emotional arousal intensities, there were no significant differences in the IOR effect among individuals with schizophrenia. This suggests that negative emotional stimuli with high arousal did not exert an additional influence on attentional capture. According to the attention disengagement theory, high arousal negative stimuli should make it more difficult for participants to disengage their attention, leading to a diminished or delayed IOR effect (49, 50). However, we propose that in Experiment 1, individuals with schizophrenia may have largely ignored the processing of stimuli at the cued location. Two main reasons may account for this finding. Experiment 1 required participants to perform a detection task on a white five-pointed star target. During the attentional disengagement phase, the emotional pictures serving as cues may have functioned primarily as spatial cues, with their emotional content being only weakly associated with the experimental task. As a result, participants likely engaged in superficial processing of the emotional cues, perceiving little to none of their emotional content (51). Second, we argue that the attentional filtering system in individuals with schizophrenia remains intact for task-irrelevant emotional information. Previous studies have shown that, within the spatial cueing paradigm, individuals with schizophrenia exhibit cueing effects comparable to those of healthy controls (52, 53). This indicates that individuals with schizophrenia are capable of selectively processing task-relevant information while filtering out irrelevant information. Therefore, in Experiment 1a, the emotional information presented at the cue location, which was irrelevant to the task, did not receive enhanced attentional processing, and no difficulties were observed during the attentional disengagement phase. In Experiment 1b, the interaction between cue validity and emotional arousal intensity was significant in the control group. Specifically, the IOR effect size under negative high arousal conditions was significantly greater than those under both negative low arousal and neutral low arousal conditions, while no significant difference emerged between negative low arousal and neutral low arousal conditions. This aligns with evidence suggesting prioritized processing of negative stimuli over neutral stimuli (54), where high arousal emotional cues receive amplified processing (55). As demonstrated, high arousal emotional cues may potentiate IOR through noradrenergic enhancement of superior colliculus-mediated IOR mechanisms, coupled with pulvinar nucleus activation for threat-signal tagging (32, 56). The pulvinar nucleus actively modulates visual attentional orienting and threat-selective processing via its extensive connectivity with visual cortices, frontoparietal networks, amygdala, and superior colliculus (57, 58).

In contrast to Experiment 1a, Experiment 2a revealed a significant interaction between cue validity and emotional arousal intensity. Specifically, there was no significant difference in the effect size of IOR between the negative low arousal and neutral low arousal conditions, suggesting that valence alone did not influence the IOR effect. However, a significant difference in the IOR effect size was observed between the negative high arousal and neutral low arousal conditions, while no significant difference was found between the negative high arousal and negative low arousal conditions. This indicates that the intensity of emotional arousal, rather than valence, is the primary factor influencing IOR in individuals with schizophrenia during the attentional redirection phase. We propose two possible explanations for this finding. First, theories of attention suggest that negative stimuli are often prioritized in processing (54), and attention is more likely to be biased toward highly arousing stimuli (59, 60). Thus, negative high arousal stimuli may capture more attention in individuals with schizophrenia. Second, while classic theories propose that individuals with schizophrenia exhibit deficits in both attending to relevant information and filtering out irrelevant information (61–63), recent studies have found that their attention may be more concentrated and narrower, exhibiting “hyperfocusing” behavior (5, 64). This overly focused attention may be directed toward task-irrelevant information, leading to impaired attentional filtering. Although emotional stimuli were presented at the target location in Experiment 2a, the task required participants to perform a detection task rather than directly process the emotional content. Thus, the emotional stimuli were essentially task-irrelevant. During the attentional reorientation phase, we propose that individuals with schizophrenia may have exhibited “hyperfocusing” on negative high arousal stimuli, leading to significant differences in the IOR effect size. This “hyperfocusing” behavior may represent one manifestation of attentional deficits in individuals with schizophrenia within the cue-back-to-fixation paradigm. In Experiment 2b, the interaction between cue validity and emotional arousal intensity in the control group was not significant, consistent with findings from previous studies (65–67), reflecting an “attentional blindness” phenomenon. The emotional motivation theory suggests that individuals are naturally drawn to positive stimuli and avoid negative stimuli, reflecting an adaptive approach-avoidance behavior. To enhance survival, individuals should allocate less attention to highly negative information, as excessive focus on negative stimuli may contribute to emotional disorders such as anxiety and depression (68, 69).

Present study mainly explains the behaviors of schizophrenia patients from a cognitive perspective. However, these explanations may have some differences from some existing models of schizophrenia, e.g. salience dysregulation (70). Salience refers to the fact that, due to the limited nature of the brain’s attentional resources, during the process of attention allocation, stimuli are prioritized based on their saliency (71). For example, we usually believe that compared with neutral and positive stimuli, negative stimuli are more salient and more likely to attract attention. Furthermore, within the same valence, stimuli with high-intensity arousal are also more likely to capture attention (32). The prioritization of processing information that is significantly relevant is believed to be related to the actions of dopamine. Altered dopamine neurotransmission will eventually lead to an impact on the selection of salient stimuli. Previous study suggests that salience dysregulation is not only a mechanism of schizophrenia but also a common mechanism of psychosis (72). According to salience dysregulation theory, high-arousal negative stimuli are more salient and may be more likely to be ignored. Our research results partially support this theory. In experiment 1a, we did not find significant difference of IOR effect size between different arousal conditions. This indicated that when high-arousal stimuli were presented at the cued location, schizophrenia patients did not show a priority of attention. Therefore, compared with healthy subjects, they may have a salience dysregulation phenomenon during the attention orientation stage. However, during the stage of attentional reorientation, this deficiency may disappear. Thus, we posited that when individuals with schizophrenia were required to respond to stimuli, their attention might be disproportionately captured. Overall, we did not examine changes in physiological indicators such as dopamine. Consequently, future research endeavors aimed at validating the aforementioned explanations necessitate the incorporation of advanced neuroimaging techniques, such as functional magnetic resonance imaging (fMRI).

Although our research initially found the modulating effect of arousal intensity and showed different patterns between schizophrenia group and control group, there were still some caveats should to be noted. Firstly, even though this study employed a simple detection task and balanced the number of men and women to a basic extent, future research still should take into account the perspective of demographic diversities to replicate the observed pattern of our results, such as age, illness duration and subgroup analyses by gender. This is because with the increase of age and duration of illness, cognitive decline may occur, and it may also become a major factor affecting the activation of emotional arousal. In addition, there may be differences in emotional sensitivity between men and women. For instance, women might be more sensitive to changes in negative emotions. Thus, we propose Male vs Female subgroup analyses for future studies. Secondly, since present study observed the inhibition of return in schizophrenia patients by changing the paradigm, the medication status of the subjects was not distinguished. However, the effects of different types of antipsychotic drugs (atypical antipsychotic drugs and typical ones) and their dosages on the attention of patients with schizophrenia may be different. This study did not take this factor into account. Future research needs to further distinguish the possible impact of these potential factors on the results. Thirdly, the *Positive and negative symptoms scale (PANSS)* (73) and comorbidities (e.g., anxiety, depression) was not measured in the present study. Since Schizophrenia is usually accompanied by psychiatric comorbidities and could influence emotional processing and attentional patterns. Future research could further distinguish the different attentional characteristics of schizophrenia patients with negative symptoms and those with positive symptoms, as well as whether there are some comorbid symptoms that have different impacts on the outcomes. Finally, although the effect sizes of *post-hoc* comparisons with Bonferroni correction for IOR achieved medium-sized effect (based on a prior sensitivity analysis), we still need to be vigilant about power limitations. For example, the 95%CI of average difference (the difference values of the IOR effect magnitude under different arousal intensities) for schizophrenia group have lager confidence intervals than control groups. This might be related to the attentional characteristics of schizophrenia patients themselves, which fluctuate more than those of control subjects.

## 5 Conclusions

In summary, the present study demonstrated that individuals with schizophrenia exhibited IOR effect when employing a cue-back-to-fixation procedure. Furthermore, this study revealed that when emotional stimuli were presented at the cue position, the schizophrenia group tended to filter out the influence of high-arousal emotional stimuli and ignore life-threatening stimuli, whereas the control group exhibited enhanced processing of emotional stimuli that led to increased IOR effect under negative high-arousal conditions, suggesting that normal participants might pay greater attention to survival-threatening stimuli. When emotional stimuli appeared at the target position, the schizophrenia group demonstrated “hyperfocusing” on negative high-arousal stimuli, showing excessive attention to life-threatening stimuli, while the control group displayed “attentional blindness” phenomenon to avoid those threatening stimuli.

## Data Availability

The raw data supporting the conclusions of this article will be made available by the authors, without undue reservation.
